# Dietary supplementation with sodium gluconate improves the growth performance and intestinal function in weaned pigs challenged with a recombinant *Escherichia coli* strain

**DOI:** 10.1186/s12917-022-03410-5

**Published:** 2022-08-06

**Authors:** Yanyan Zhang, Dan Yi, Haiwang Xu, Zihan Tan, Yuxuan Meng, Tao Wu, Lei Wang, Di Zhao, Yongqing Hou

**Affiliations:** Hubei Key Laboratory of Animal Nutrition and Feed Science; School of animal science and nutrition engineering, Wuhan Polytechnic University, Wuhan, 430023 China

**Keywords:** Weaned pig, Growth performance, Sodium gluconate, Recombinant *Escherichia coli* strain, Intestinal function

## Abstract

**Background:**

The purpose of this research is to determine the effects of sodium gluconate (SG) on the growth performance and intestinal function in weaned pigs challenged with a recombinant *Escherichia coli* strain expressing heat-stable type I toxin (STa).

**Results:**

Pigs (*n* = 24, 21 days of age) were randomly allocated to three treatments: Control group (pigs were fed basal diet), STa group (pigs were fed basal diet and challenged with a recombinant *E. coli* strain expressing STa), and SG group (pigs were fed basal diet supplemented with 2500 mg/kg sodium gluconate and challenged with a recombinant *E. coli* strain expressing STa). The trial period lasted for 15 days. On days 12 and 13, pigs in the STa and SG groups were orally administered with the recombinant *Escherichia coli* strain, while those in the control group were orally administered with normal saline at the same volume. On day 15, blood, intestinal tissues and colonic contents were collected for further analysis. Results showed that dietary SG supplementation had a tendency to increase average daily gain, and reduced (*P* < 0.05) feed to gain ratio, plasma glucose concentration, and mean corpuscular hemoglobin concentration as compared with control group on days 0-10 of trial. Additionally, dietary SG supplementation attenuated(*P* < 0.05) the morphological abnormalities of small intestinal and the increase of the number of eosinophils in blood of pigs challenged with the recombinant *Escherichia coli* strain on day 15 of trial. Compared with control group, diarrhea rate and the number of eosinophils in blood and the concentrations of malondialdehyde in the jejunum were increased (*P* < 0.05). The height, width and surface area of the villi of the duodenum, the width and surface area of villi of jejunum and the height and width of villi of ileum were decreased (*P* < 0.05) in pigs challenged with the recombinant *Escherichia coli* strain in the STa group compared with those in control group on day 15 of trial. However, these adverse effects were ameliorated (*P* < 0.05) by SG supplementation in the SG group on day 15 of trial. Furthermore, dietary SG supplementation could reduce (*P* < 0.05) the total bacterial abundance in the colon, but SG did not restore the recombinant *Escherichia coli*-induced microbiota imbalance in colon.

**Conclusions:**

In conclusion, dietary supplementation with SG could improve piglet growth performance and alleviate the recombinant *Escherichia coli*-induced intestinal injury, suggesting that SG may be a promising feed additive for swine.

## Background

The intestine, as the first line of defense against infections, is an important immune organ in the host, which is always exposed to a large number of toxins and antigens [1]. The mucosal barrier is the host first line of defense against the external environment [2]. Since many pathogens invade the host through the intestinal mucosa, the damage of the intestinal mucosal barrier will cause inflammation and sepsis [3, 4]. Weaning stress can be easily triggered due to poor digestion and stress resistance in weanling pigs, which leads to significant changes in intestinal morphology including severe villus atrophy, accelerated crypt cell division, and significant reduction in villus ratio, severely affecting the normal function of pig intestine [5, 6]. The intestinal structural damage induced by weaning stress causes reduced appetite, digestive disorders, diarrhea, slow growth, low feed utilization, poor mental state and appearance performance, which elicits great losses to pig industry [1, 7–9].

Nutritional measures are extensively employed to reduce weaning stress except controlling environmental conditions [10, 11]. Although in-feed antibiotics, high zinc, and high copper can effectively prevent pig diarrhea, widespread concerns have arisen on the antibiotic resistance and environmental pollution problems [12, 13]. Therefore, safe and environmentally friendly feed additives, such as probiotics, prebiotics, glutamine, antibacterial peptides and acidifiers, are gradually being valued in porcine production [14–17]. So far, many new feed additives have been developed, which can effectively improve growth performance and reduce diarrhea by improving intestinal function and immunity in the pigs [18–20]. Although many valuable feed additives have been studied, in order to promote the healthy development of the pig industry, more effective feed additives need to be developed. Because there are various problems that plague the healthy growth of piglets. The development of feed additives for different problems of piglet healthy growth will be more conducive to the efficient development of the pig industry. Meanwhile, the research reports on the effects of different potential feed additives can be used as a reference for other researchers, which will help them study more potential effective feed additives. Therefore, the effects of more potential feed additives on the growth performance of piglets need to be continuously studied. Sodium gluconate (SG) is different from the feed additives on the market including probiotics, amino acids and plant extracts, which is an organic compound. As a potential feed additive, SG has stable properties and is easy to industrialize. However, there is limited research on SG as a potential feed additive. Therefore, it is meaningful to study SG as a feed additive on the performance of piglets.

SG is also called gluconic acid, sodium salt, the chemical formula for which is C_6_H_11_NaO_7_ (Molecular weight: 218.14). SG is a beige solid organic compound that is chemically stable at room temperature. SG can effectively prevent hyponatremia syndrome, regulate the acid-base balance of the body and restore normal nerve activity. SG, as a new food additive that has attracted much attention, which has the advantages of abundant raw materials, better stability and so on [21]. The supplementation of SG in feed is safe and stable, and SG is easily absorbed in the intestine. In addition, SG can improve feed quality by preventing protein degradation and masking undesirable bitterness and astringency [22–24]. Previous studies have shown that dietary SG supplementation (2% SG contained in the diets, w/w) could improve the growth performance, the utilization of phytate phosphorus and the tibia ash content in chicks [25]. Moreover, SG (0.5% SG contained in the diets, w/w) could improve fecal odor index, hydrogen sulfide and ammonia production, enhance the growth of animals and prevent the *Escherichia coli* (*E. coli*) infection [26]. However, studies on dietary supplementation of SG to improve the growth performance of animals infected with pathogens are still very limited. Because the invasion of pathogens into intestinal epithelium is complex and the heat-stable type I (STa), one of enterotoxins produced by enterotoxigenic *Escherichia coli*, can impair intestinal function, our lab has established a pig diarrhea model induced by a recombinant *E. coli* strain expressing STa (LMG194-STa strain) to study the ability of feed additives to resist pathogenic infection in piglet intestine [27]. Therefore, the effects of dietary SG supplementation on the growth performance and intestinal function in pigs challenged by recombinant STa-expressing *E. coli* will be studied in this study.

## Results

### Growth performance

**Fig. 1:**
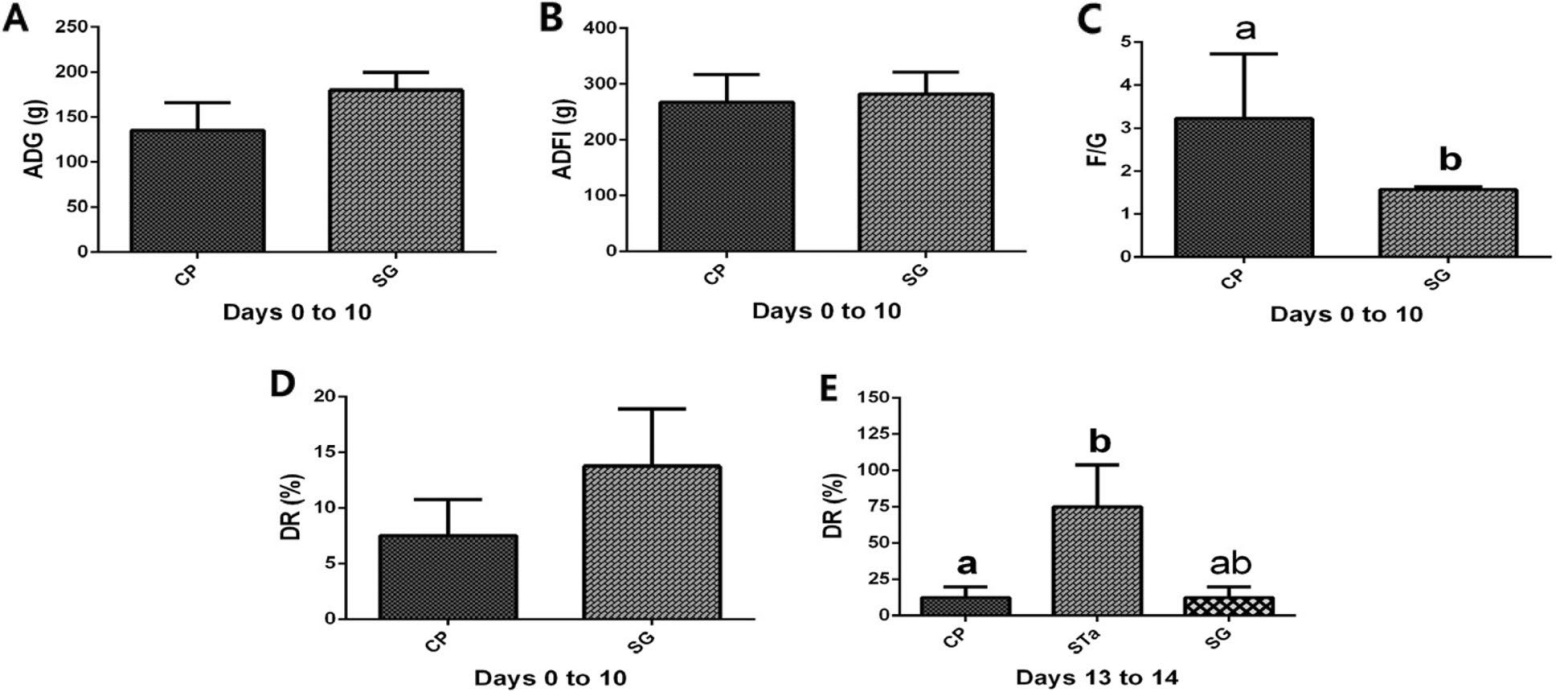
Effects of SG on the growth performance of piglets. The ADG (**A**), ADFI (**B**), F/G (**C**) and DR (**D**) on days 0-10 (before infection); The DR (**E**) on days 13-14 (post infection). CP: Control group; STa: STa group; SG: SG + STa group. Values are means and SD, *n* = 8. ^a, b^, Values within a column not sharing a common superscript letter indicate significant difference at *P* < 0.05

During days 0-10 of the trial, dietary supplementation with SG had a tendency to increase the average daily gain (ADG) (Fig. 1A). Dietary supplementation with SG did not affect the average daily feed intake (ADFI) (Fig. 1B). Dietary supplementation with SG reduced (*P* < 0.05) the feed to gain (F/G) ratio (Fig. 1C). Dietary supplementation with SG did not affect the diarrhea rate (DR) (Fig. 1D). On day 15 of the trial, the recombinant *E. coli* challenge increased (*P* < 0.05) the DR (Fig. 1E) of pigs in STa group. Dietary supplementation with SG had a tendency to reducethe increase inDR of pigs challenged with recombinant *E. coli* in SG group (Fig. 1E).

### Blood biochemical and hematological parameters

**Fig. 2:**
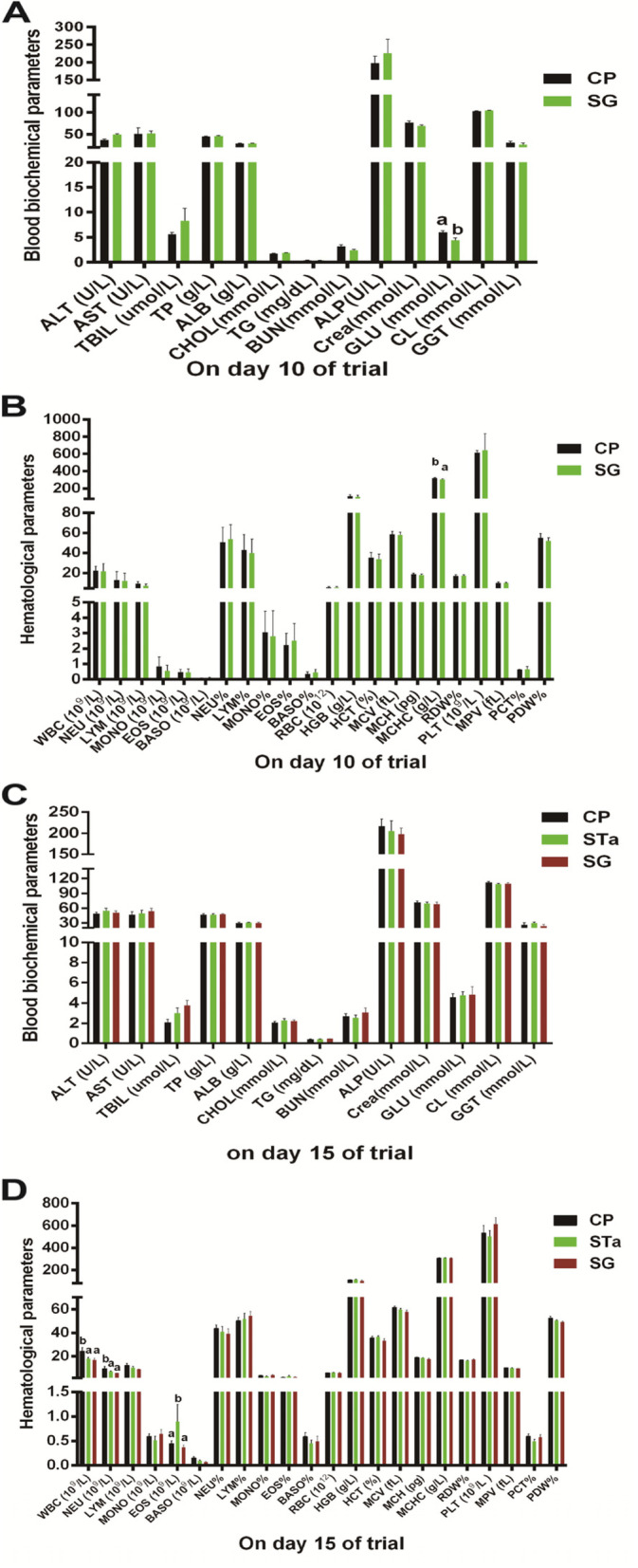
Effects of SG on blood biochemical and hematological parameters in pigs. The blood biochemical parameters (**A**) and hematological parameters (**B**) on day 10 (before infection); blood biochemical parameters (**C**) and hematological parameters (**D**) on day 15 (post infection). CP: Control group; STa: STa group; SG: SG + STa group. Values are mean and SD, *n* = 8. ^a, b^, Values within a column not sharing a common superscript letter indicate significant difference at *P* < 0.05

The biochemical and hematological parameters are presented in Fig. 2. On day 10 of the trial, the concentration of blood glucose (Fig. 2A) and the mean corpuscular hemoglobin concentration (MCHC) (Fig. 2B) were decreased (*P* < 0.05) by SG supplementation. On day 15 of the trial, the recombinant *E. coli* challenge did not affect blood biochemical parameters (Fig. 2C). However, the recombinant *E. coli* challenge reduced (*P* < 0.05) the number of white cells and neutrophils in the blood, while increasing (*P* < 0.05) the number of eosinophils (EOS) in the blood (Fig. 2D) of pigs. Dietary supplementation of SG could restore (*P* < 0.05) the number of eosinophils in pigs challenged with the recombinant *E. coli* to normal levels (Fig. 2D).

### Effects of SG supplementation on intestinal morphology

**Fig. 3:**
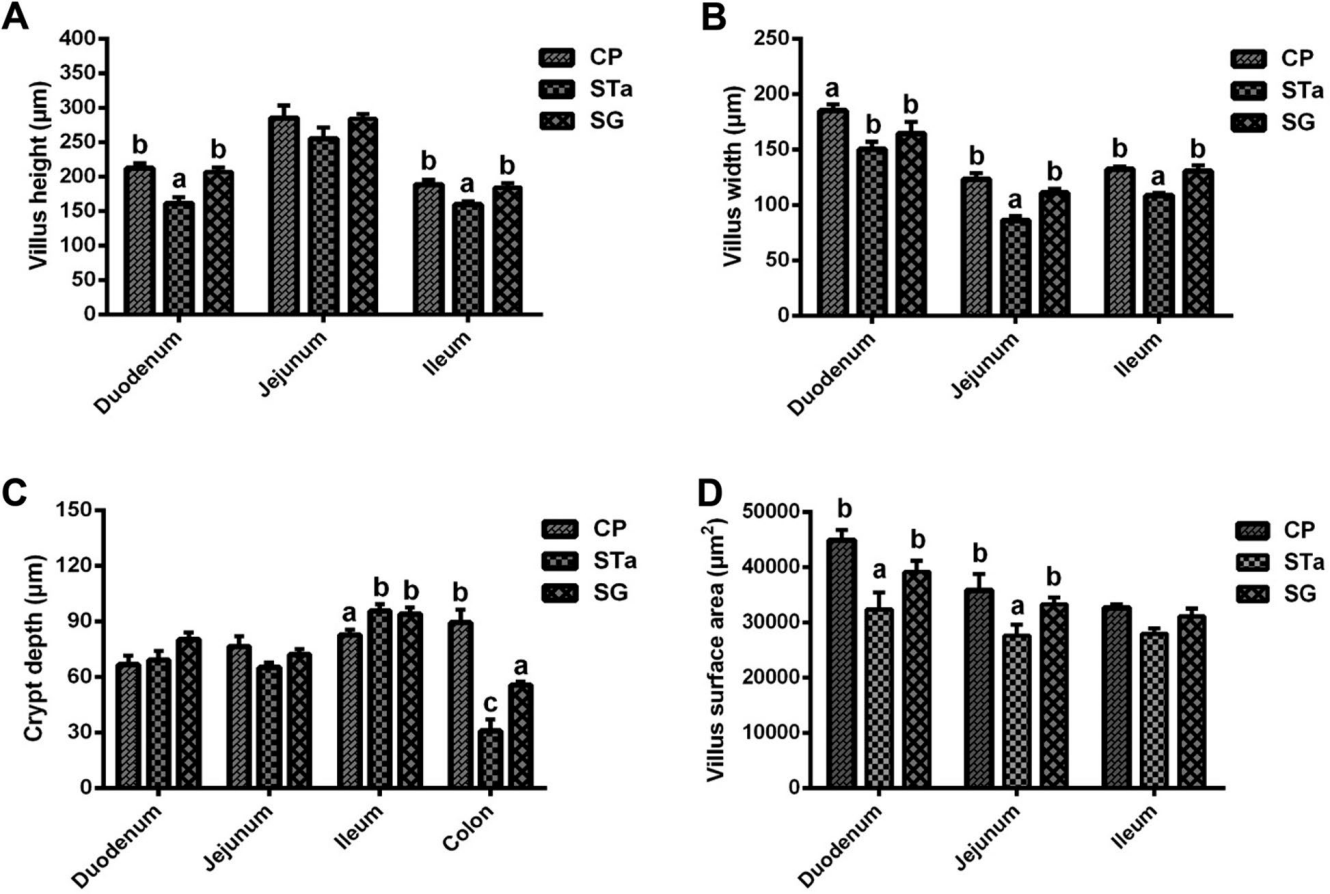
Effects of SG supplementation on intestinal morphology of pigs. CP: Control group; STa: STa group; SG: SG + STa group. Values are mean and SD, *n* = 8. ^a, b, c^, Values within a column not sharing a common superscript letter indicate significant difference at *P* < 0.05

As illustrated in Fig. 3, the recombinant *E. coli* challenge reduced (*P* < 0.05) the villus height of the duodenum and ileum, which could be mitigated (*P* < 0.05) by dietary supplementation of SG (Fig. 3A). The recombinant *E. coli* challenge decreased (*P* < 0.05) the villus width of the duodenum, jejunum and ileum, while dietary supplementation of SG could alleviate (*P* < 0.05) the reduction in the villus width of the jejunum and ileum in pigs challenged with the recombinant *E. coli* strain (Fig. 3B). The recombinant *E. coli* challenge increased (*P* < 0.05) the ileal crypt depth, but decreased (*P* < 0.05) the colonic crypt depth (Fig. 3C). However, dietary SG supplementation could inhibit (*P* < 0.05) the decrease in colonic crypt depth induced by the recombinant *E. coli* challenge (Fig. 3C). Additionally, recombinant *E. coli* challenge induced (*P* < 0.05) the reductions in the villus surface area of the duodenum and jejunum, which could be restored (*P* < 0.05) by dietary supplementation of SG (Fig. 3D).

### Effects of SG supplementation on intestinal redox status

**Fig. 4:**
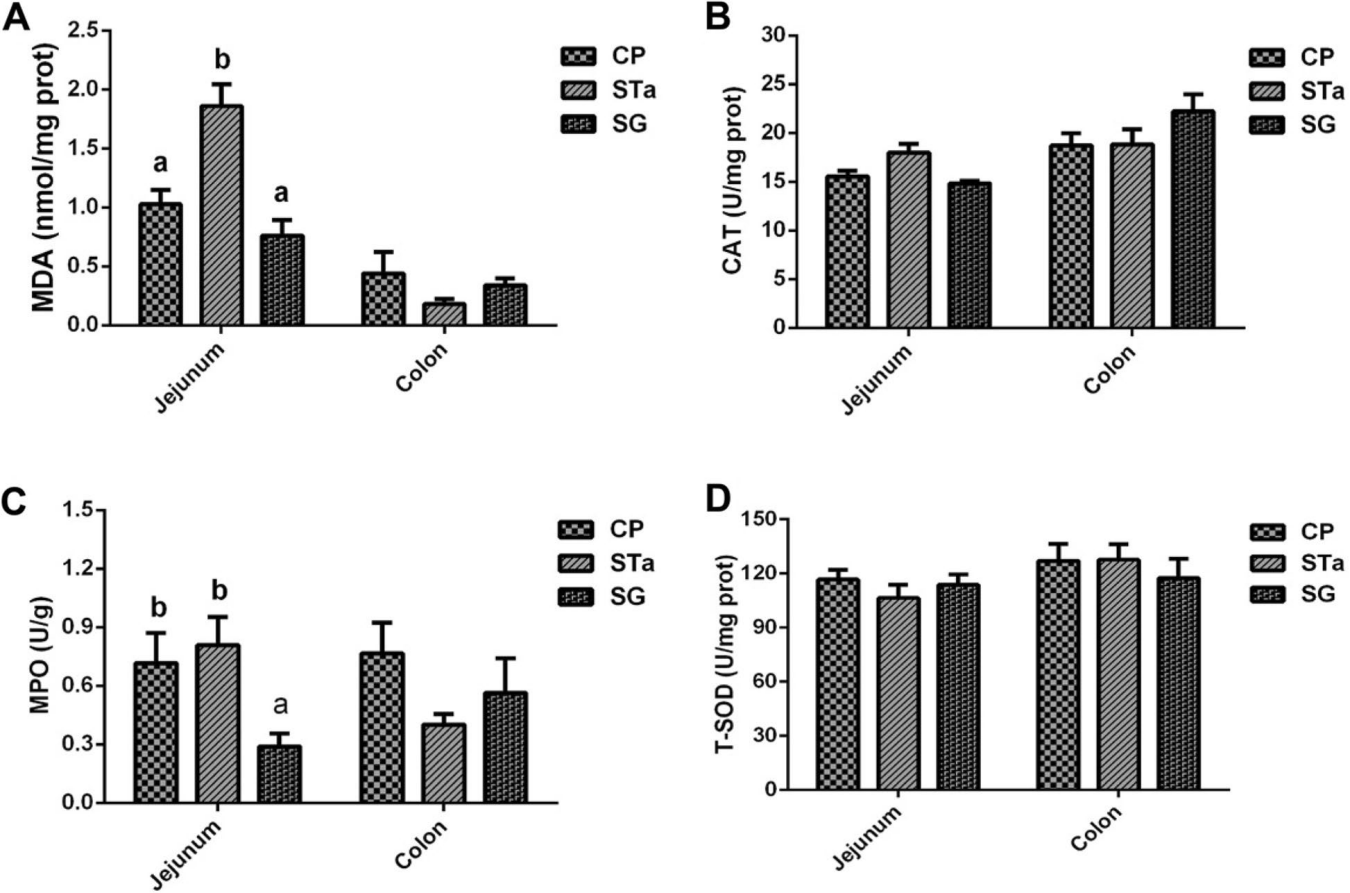
Effects of SG on intestinal redox status in piglets. CP: Control group; STa: STa group; SG: SG + STa group. Values are mean and SD, *n* = 8. ^a, b^, Values within a column not sharing a common superscript letter indicate significant difference at *P* < 0.05

As shown in Fig. 4, compared with the pigs of the control group, the recombinant *E. coli*-challenged pigs exhibited an increase (*P* < 0.05) in MDA concentration in the jejunum (Fig. 4A). Dietary supplementation of SG decreased (*P* < 0.05) the concentration of MDA in the jejunum in SG group (Fig. 4A). The recombinant *E. coli*-challenged or dietary supplementation of SG pigs did not exhibited difference in CAT activity in the jejunum and colon (Fig. 4B). Dietary supplementation of SG decreased (*P* < 0.05) the activity of MPO in the jejunum of pigs challenged with recombinant *E. coli* in SG group (Fig. 4C). The recombinant *E. coli*-challenged or dietary supplementation of SG pigs did not exhibited difference in T-SOD activity in the jejunum and colon (Fig. 4D).

### Abundance of intestinal bacteria

**Fig. 5:**
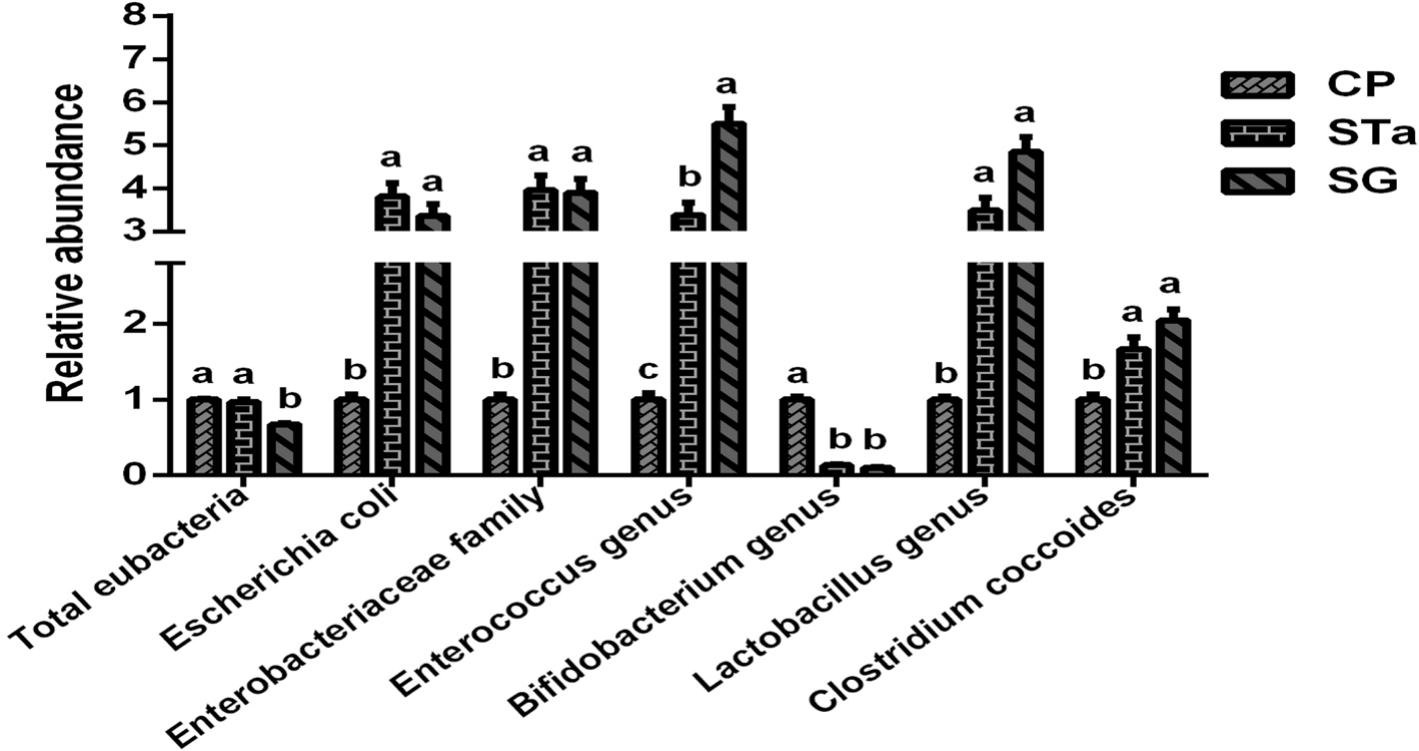
Effects of dietary SG supplementation on colon bacteria of pigs after recombinant *E. coli* challenge. CP: Control group; STa: STa group; SG: SG + STa group. Values are mean and SD, *n* = 8. ^a, b, c^, Values within a column not sharing a common superscript letter indicate significant difference at *P* < 0.05

Compared with the control group, recombinant *E. coli* infection increased (*P* < 0.05) the number of *E. coli*, *Enterobacteriaceae* family, *Enterococcus* genus, *Lactobacillus* genus and *Clostridium coccoides*, but reduced (*P* < 0.05) the number of *Bifidobacterium* genus in the colon of pigs. Dietary SG supplementation did not restore the changes in the number of *E. coli*, *Enterobacteriaceae* family, *Enterococcus* genus, *Lactobacillus* genus, *Clostridium coccoides* and *Bifidobacterium* genus caused by recombinant *E. coli* challenge. However, dietary SG supplementation reduced (*P* < 0.05) the number of the total bacterial abundance in the colon of pigs in SG group (Fig. 5).

## Discussion

In order to cope with the impaired growth, diarrhea and other diseases in weaning pigs, antibiotics are widely used as feed additives [28, 29]. However, long-term use of antibiotics in feed leads to antibiotic resistance, which disrupts the microecological balance in the intestines, induces secondary infections, and makes the treatment of bacterial infections more difficult. So far, antibiotic resistance has received widespread public concern [30, 31]. Antibiotics in feed are considered a risk factor for animal food safety in the world [32, 33]. China, the European Union, South Korea, the United States and other countries ban the addition of preventive antibiotics to feed [34]. To address the problem of antibiotic resistance, many potential antibiotic alternatives have been studied. These alternatives focus on reducing piglet diarrhea, lowering the crude protein and increasing the crude fiber in the piglet’s diet [35, 36]. In recent years, a variety of additives that may replace antibiotics have been extensively studied, including prebiotics, plant extracts, acidifiers, etc. [37]. In this study, we found that dietary SG supplementation had a tendency to increase the ADG (Fig. 1A) of pigs during days 0-10 of the trial. This result was consistent with that of Kang et al. (2014), who reported that dietary supplementation with 0.5% proline had a tendency to increase ADG [38]. Previous study has reported that *Enterococcus faecium* R1 can improve piglet growth performance by significantly reducing DR and F/G [18]. In agreement with previous research results [18], our findings exhibited that dietary SG supplementation reduced (*P* < 0.05) the F/G (Fig. 1C) during days 0-10 of the trial. Although dietary SG supplementation had no effect on the DR (Fig. 1D) of pigs before infection, it had a tendency to reduce the increase in DR (Fig. 1E) in pigs challenged with recombinant *E. coli*. These results suggested that dietary SG supplementation could improve the growth performance of piglets by increasing ADG and reducing F/G and exert beneficial effect in combating diarrhea in piglets caused by *E. coli* infection. Moreover, SG may have similar functions with *Enterococcus faecium* R1 and proline in improving the growth performance of piglets.

Although dietary SG supplementation improved the growth performance of pigs including the increased ADG and reduced F/G, it is remained unclear whether dietary supplementation of SG could enhance the ability of pigs to resist pathogenic infections. So far, weaned pig diarrhea induced by pathogenic microorganisms has greatly restricted the healthy development of pig farming and causes tremendous economic losses to the pig industry every year [39]. As a major cause of piglet diarrhea, enterotoxigenic *E. coli* colonizes the small intestine and then expresses the heat-stable enterotoxin. Pig diarrhea caused by the heat-stable enterotoxin can last for 3-5 days. Pig infected with enterotoxigenic *E. coli* will have mild diarrhea in the early stage and severe diarrhea causing dehydration in the later stage. In order to develop eco-friendly feed additives against enterotoxigenic *E. coli* infection, suitable and reliable animal models of infection are required. In previous studies, naturally infected pigs were often used as a model to evaluate the effectiveness of potential eco-friendly feed additives [40–43]. In the latest research, a piglet diarrhea model induced by recombinant *E. coli* was established, which can be used for nutritional and mechanistic studies of intestinal dysfunction [27]. Therefore, in this study, a piglet diarrhea model induced by recombinant *E. coli* was used to determine the ability of the dietary SG supplementation to resist enterotoxigenic *E. coli* infection in pigs.

Early weaning of pigs will cause changes in blood indicators, which can reflect the status of health, the level of metabolism, and cell permeability [44]. It is worth noting that dietary SG supplementation can reduce (*P* < 0.05) blood glucose (Fig. 2A) and MCHC (Fig. 2B) levels of pigs, but their values are still within the normal range. White blood cells play an important role in the host resistance to pathogen invasion, which are regarded as the important defense lines of the host defense system. When the host is infected by pathogens, the number and proportion of white blood cells will be changed [45]. Eosinophils quickly swallow pathogens that invade the host, such as bacteria and parasites. Neutrophils play a crucial part in the host innate immune system, which can engulf and kill pathogens [46]. In this study, the number of eosinophils was increased (*P* < 0.05), but the number of white blood cells and neutrophils was reduced (*P* < 0.05) in the STa group (Fig. 2D). Contrary to the previous research that dietary *Lactobacillus rhamnosus* LB1 supplementation significantly increased the number of eosinophils (*P* < 0.05) in pigs challenged with *E. coli* [47]. Our result showed that dietary SG supplementation attenuating the increase of the number of eosinophils (*P* < 0.05) in pigs challenged with recombinant *E. coli* (Fig. 2D). These results suggested that recombinant *E. coli* infection could cause immunological stress and dietary supplementation of SG could alleviate the host immunological stress.

The small intestine is the most important nutrient absorption organ in the animal body. Early weaning leads to mucosal atrophy, villus shortening, and crypt deepening in the small intestine [1, 48]. The surface area and width of villi reflect the ability of the small intestine to absorb nutrients. Some studies showed that the digestion and absorption of nutrients was closely related to the morphology of intestinal villi [49]. Under normal physiological conditions, the small intestine can effectively absorb nutrients. When *E. coli* infection impaired the mucosal morphology, the small intestinal dysfunction occurred [50]. Previous study reported that dietary N-Acetylcysteine supplementation improved the decrease of villus height in piglets challenged with porcine epidemic diarrhea virus [6]. In this study, these results showed that dietary SG supplementation not only exhibited an increase (*P* < 0.05) in the villus height of the duodenum and ileum (Fig. 3A), but also exhibited the elevation (*P* < 0.05) in the villus width of the duodenum, jejunum and ileum (Fig. 3B) and the increase (*P* < 0.05) in the villus surface area of the duodenum and jejunum in piglets challenged with recombinant *E. coli.* (Fig. 3D). These results suggested that dietary SG supplementation exhibited better effectsin repairing small intestinal injury caused by pathogenic microorganisms.. It is also suggested that dietary SG supplementation can reduce the increase of diarrhea in piglets challenged with recombinant *E. coli* by improving the small intestinal morphology.

As an active protease, SOD is a scavenger of oxygen free radicals in the body, which can prevent lipid peroxidation from damaging the structure and function of cell membranes. It is an important antioxidant enzyme in the organism, which can remove various metabolic wastes in tissues and cells, and maintain the antioxidant capacity of the host [51]. MDA is the final metabolite of lipid peroxidation in the body through ROS chain reaction. The degree of membrane oxidation can be determined based on the content of MDA, thereby assessing the degree of damage to the cell membrane [52]. CAT, an anti-oxidative enzyme, can catalyze the reduction of H_2_O_2_ to water [51–53]. Myeloperoxidase (MPO), as a rich granule enzyme, is a heme-containing peroxidase expressed mainly in neutrophils, which can catalyze the production of potent ROS [53]. MPO activity is related to the normal function and activation of neutrophils. Elevated levels of MPO are also associated with pathology such as coronary artery disease and endemic arsenic poisoning. In agreement with previous research results that dietary N-Acetylcysteine supplementation significantly reduced the increase of the content of MDA and the activity of MPO in jejunum in piglets challenged with β-conglycinin [54]. Our results showed that dietary SG supplementation reduced (*P* < 0.05) the content of MDA (Fig. 4A) and the activity of MPO (Fig. 4C) in the jejunum of pig challenged with recombinant *E. coli.* These results suggested that dietary SG supplementation could protect pig jejunal mucosae from oxidative stress by decreasing the content of MDA and the activity of MPO.

Some intestinal microbes can improve the host immune function and enhance the ability to resist infection [55]. Once intestinal flora homeostasis is disrupted, a large number of pathogenic bacteria will proliferate and damage intestinal mucosal, causing diarrhea and even sepsis. The intestinal mucosa and epithelial cells constitute the first biological barrier, which regulates the balance of the inherent flora and resists pathogens [4]. *Lactobacillus* genus can regulate the immune function development of pigs and resist inflammation caused by foreign antigens [56]. *Lactobacillus* genus can also enhance the activity of macrophages, and increase the number of WBC and serum protein content to improve the host immunity [57]. *Bifidobacterium* genus is an anaerobic gram-positive bacteria, which will be decreased in the intestine with age and can effectively treat diarrhea. *Bifidobacterium* genus could activate the macrophages, which functions in the host innate immune response. Metabolites of *Bifidobacterium* genus, such as protein and hydrogen peroxide, can inhibit or even kill pathogenic bacteria [58]. *Clostridium coccoides* is an anaerobic gram-positive bacillus, which can cause enteritis. Although *Enterococcus* genus is considered to have a positive role in cheese technology, it is regarded as an unreliable bacteria. This study showed that the recombinant *E. coli* infection caused the elevation (*P* < 0.05) in the number of *E. coli*, *Enterobacteriaceae* family, *Enterococcus* genus, *Lactobacillus* genus and *Clostridium coccoides* in the colon of pigs (Fig. 5), leading to a significant imbalance of the colon flora. Dietary SG supplementation cannot change the flora imbalance caused by pathogenic microorganisms, which was in agreement with previous research results that dietary puerarin supplementation cannot change the flora imbalance in the colon caused by pathogenic microorganisms [32]. Dietary puerarin supplementation significantly reduced the total bacterial abundance in the colon [32]. In this study, dietary SG supplementation reduced (*P* < 0.05) the total bacterial abundance in the colon. (Fig. 5). These results showed that recombinant *E. coli* challenge significantly increased the abundance of the dominant flora including *Escherichia coli*, *Enterobacteriaceae* family, *Enterococcus genus*, *Lactobacillus genus* and *Clostridium coccoides*. The dietary SG supplementation cannot affect the increase of the abundance of the dominant flora. However, the dietary SG supplementation significantly reduced the total bacterial abundance in the colon. These results suggested that SG may lead to a decrease in the total bacterial abundance by reducing the abundance of non-dominant flora in the colon.

## Conclusions

In conclusion, dietary SG supplementation could improve the growth performance of pigs and alleviated the recombinant *E. coli-*induced intestinal injury in pigs. This research provides an important implication for the application of SG in the pig industry.

## Materials and methods

### Animals and experimental design

The animal experiment was approved by the Animal Care and Use Committee at Wuhan Polytechnic University (No. WPU202011003). Twenty-four healthy female crossbred pigs (Duroc×Landrace×Yorkshire) with similar body weight (5.56 Kg) were weaned at 21 days of age. All pigs were housed individually in stainless steel metabolic cages (1.20 × 1.10 m^2^), maintained at an ambient temperature of 22-25 °C in an environmentally controlled room by air-conditioning and acclimatized for 3 days. In order to avoid diarrhea caused by overfeeding in weaned pigs, this study adopted the restrict feeding. Pigs were fed 5 times a day at 8:00, 12:00, 15:00, 18:00, and 21:00, respectively. The corn- and soybean meal-based diet was formulated to meet National Research Council (NRC, 2012)-recommended requirements for all nutrients (Table 1) [18]. All piglets were randomly divided into three treatment groups (8 pigs per group): (1) control group, (2) STa group, and (3) SG group, which were fed the basal diet during the 3-day adaptation period. At 24 days of the age, Pigs in the control group and the STa group were fed a basal diet, and those in the SG group were fed the basal diet supplemented with 2500 mg/kg SG (Sigma-Aldrich-S2054). During days 1-10 of the trial, the growth performance of pigs in each group was analyzed by recording the body weight, the feed intake and diarrhea. To determine average daily gain (ADG), average daily fed intake (ADFI) and feed/gain ratio (F/G), individual piglets were weighed on days 1 and 10 of the trial, daily fed intake was the actual daily feed intake of weaned piglets. ADG (Kg/day) = gain (Kg, 8 piglets per treatment group)/10. ADFI (Kg/day) = total feed intake (Kg, 8 piglets per treatment group)/10. F/G (%) = total feed intake (Kg)/ gain (Kg, 8 piglets per treatment group) × 100. To determine the diarrhea rate, individual piglets were examined for diarrhea four times per day. Diarrhea was quantified by the following equation for each pig. Diarrhea rate (DR) (%) = total diarrhea incidences/8 × 100. The recombinant STa-expressing *E. coli* strain was grown at 37 °C in Luria-Bertani (LB) medium. After 8 hours of cultivation, the recombinant *E. coli* (OD_600_ = 0.8) strains were collected by centrifuging the bacterial cultures at 5000 rpm for 10 min at room temperature. Subsequently, the bacterial cells were suspended in physiological saline (1.0 × 10^9^ CFU/mL). On days 12 and 13 of the trial, pigs in the STa and the SG groups were orally administered with the recombinant STa-expressing *E. coli* strain (10 mL) twice a day. Pigs in the control group were orally administered with the same volume of saline. During days 12-15 of the trial, the effects of SG on the growth performance in pigs challenged with the recombinant *E. coli* strain were analyzed by recording the feed intake, diarrhea, the body weight and average daily fed intake. On day 15 of the trial, all pigs were killed and dissected under anesthesia with an intravenous injection of pentobarbital sodium (50 mg/kg BW) to obtain intestinal tissues and contents by the previously described with minor modifications [33]. First, the abdominal cavity of the slaughtered pig was immediately opened with a scalpel to expose the whole gastrointestinal tract. Subsequently, the intestine was separated from the mesentery by using surgical scissors and immediately the separated intestine was placed on a frozen stainless steel tray. Finally, the 5-cm and 10-cm intestinal segments were separately collected from the distal duodenum, mid-jejunum, mid-ileum, and mid-colon. For morphological measurements, the 5-cm intestinal segments were washed gently with ice-cold normal saline and then fixed with 4% paraformaldehyde. The contents were carefully flushed with ice-cold normal saline from the 10-cm segments opened longitudinally. Mucosa was scraped and collected by using a sterile glass microscope slide at 4 °C from the 10-cm segments opened longitudinally without contents. The intestinal mucosae and contents collected were rapidly frozen in liquid nitrogen and stored at − 80 °C until analysis.

**Table 1:** Ingredients and contents of energy and nutrients of the basal diet (air-dry basis)

Items	Content
Ingredients (%)	
Corn (DE 14.27 MJ/kg, CP 8.7%)	61.88
Soybean meal l (DE 13.5 MJ/kg, CP 40%)	21.98
Wheat Middling (DE 13.4 MJ/kg, CP 13%)	4.00
Dried whey (CP 12%)	3.00
Fish meal (CP 66%)	3.00
Soy protein concentrate (CP 65%)	1.50
CaHPO_4_	1.25
Premix^a^	1.00
Limestone (CaCO_3_ > 35%)	0.69
NaCl	0.30
Acidifier (Citric acid > 99%)	0.30
Soybean oil	0.50
L-Lysine HCl (78.8% lysine) Sodium gluconate^b^	0.25 0.25
Choline chloride	0.20
Mould inhibitor (Calcium propionate > 30%)	0.10
DL-Methionine (99% methionine	0.05
Nutrients composition	
Digestible energy^b^ (MJ/kg)	14.22
Crude protein (%)^c^	20.9
Total threonine (%)^c^	0.74
Total methionine (%)^c^	0.30
Total lysine (%)^c^	1.15
Total tryptophan (%)^c^	0.21
Total calcium (%)^c^	0.70
Total phosphorus (%)^c^	0.60
Available phosphorus (%)^b^	0.32

^a^Premix provided the following amounts of vitamins and trace minerals per kilogram of the complete diet: ferrum, 100 mg (FeSO_4_·H_2_O); copper, 150 mg (CuSO_4_·5H_2_O); manganese, 40 mg (MnSO_4_·5H_2_O); zinc, 100 mg (ZnSO_4_·7H_2_O); iodine, 0.5 mg (KI); selenium, 0.3 mg (Na_2_SeO_3_·5H_2_O); vitamin A acetate, 3.66 mg; cholecalciferol, 0.10 mg; DL-α-tocopheryl acetate, 36.4 mg; menadione, 4 mg; thiamin, 6 mg; riboflavin, 12 mg; pyridoxine, 6 mg; cyanocobalamin, 0.05 mg; biotin, 0.2 mg; folic acid, 2 mg; niacin, 50 mg; D -calcium pantothenate, 25 mg

^b^Calculated value

^c^Analyzed value

### Blood sample collection

On days 10 and 15 of the trial, blood was collected from the anterior vena cava of each piglet by using 10 mL heparinized vacuum tubes. After standing at room temperature for 15 min, the blood samples were centrifuged at 3500 rpm for 15 min to separate plasma, which was further aliquoted in centrifuge tubes and stored at -80 °C until analysis. Plasma biochemical parameters (including alanine transaminase, ALT; aspartate transaminase, AST; total bilirubin, TBIL; total protein, TP; albuminuria, ALB; cholesterol, CHOL; triglyceride, TG; blood urea nitrogen, BUN; alkaline phosphatase, ALP; creatinine, Crea; glucose, GLU; chloride, CL; glutamyl transferase, GGT) were measured with corresponding kits using a Hi-tachi 7020 Automatic Biochemical Analyzer (Hitachi, Tokyo, Japan). Theses kits used for plasma biochemical parameters were purchased from Hitachi Hi Tech Co., Ltd. Before starting to measure samples, the relevant parameters of different plasma biochemical parameters have been input into the system according to the instructions. Automatic blood analyzer (ADVIA® 2120/2120i, Siemens Healthcare Diagnostics Inc.) was used to measure hematological parameters. The test tube containing the sample was gently shaken to fully mix the blood sample, and then placed the test tube under the sampling needle. Then, the instrument was started running and analysis results were collected.

### Measurement of intestinal morphology

To measure intestinal morphology, the paraformaldehyde-fixed 5-cm segments of duodenum, jejunum, ileum, and colon was taken, respectively. 5-cm segment of duodenum was collected from the front of the distal 15-cm of the duodenum. 5-cm segments of jejunum, ileum and colon were collected from the front of the middle 15 cm of the jejunum, ileum and colon, respectively. Subsequently, the segments of duodenum, jejunum, ileum and colon were separately dehydrated and embedded in paraffin. The intestinal sections were cut and then stained with hematoxylin and eosin stain. Intestinal morphology was measured by using a light microscope (Leica, Solms, Germany) as previously described with minor modifications [15]. Crypt depth (the distance from the crypt mouth to the base), villus width (the distance of the widest villi) and height (the distance from the villus tip to the crypt mouth) were measured using a linear ocular micrometer with a computer-assisted morphometric system (Leica, Solms, Germany). Villi and crypts that were only vertically oriented were measured. The surface area of the villus is equal to the height of the villus multiplied by the width of the villus.

### Determination of the redox status in the intestinal mucosae

In order to obtain the mucosa, the collected intestinal segment was cut longitudinally, exposing the inside of the intestine. Then the cut intestines were spread flat on ice, with the intestine inner side facing up, and a thin glass slide was use to scrape the mucosa from the inside of the intestine. The collected mucosa was wrapped in tin foil and then frozen in liquid nitrogen until use. After grinding the frozen intestinal mucosa samples, the samples were well mixed in a nine-fold volume of pre-cooled saline. The mixture was then centrifuged at 3500 rpm for 15 min at 4 °C, and the supernatant was collected and frozen at -80 °C. Total superoxide dismutase (T-SOD), catalase (CAT), myeloperoxidase (MPO) and malondialdehyde (MDA) in the intestinal mucosae were determined by using commercially available kits including Total Superoxide Dismutase (T-SOD) assay kit (Hydroxylamine method), Catalase (CAT) assay kit (Ultraviolet), Myeloperoxidase assay kit, and Malondialdehyde (MDA) assay kit (TBA method) (Nanjing Jiancheng Bioengineering Institute, Nanjing, China).

### Determination of intestinal microflora in colon digesta

The colon was placed on ice, and then a segment of intestine containing digesta was taken. The digesta was taken out as described above, placed in a sterile tube, stirred evenly and then stored in liquid nitrogen for the determination of microorganisms in the colon. The determination method for the number of total eubacteria, *E. coli*, *Enterobacteriaceae* family, *Enterococcus* genus, *Clostridium coccoides*, *Lactobacillus* genus, and *Bifidobacterium* genus in colon chyme was described briefly as follows. First of all, the intestinal contents were evenly ground in liquid nitrogen, and then 180-220 mg of uniformly ground intestinal contents were weighed and placed in a 1.5 mL centrifuge tube. Subsequently, the microbial DNA in colon chyme was extracted by using QIAamp™Fast DNA Stool Mini Kit (Cat. No.51604) as described by Yi et al. [29]. Finally, the aforementioned microorganisms in the colon chyme were determined by quantitative RT-PCR (qRT-PCR). Microbial samples were normalized by using the average cycle threshold (Ct) of 16S rDNA [29, 30] as a reference. Results were analyzed by using the 2 ^−ΔΔCt^ method [29, 30]. Primer sequences used for qRT-PCR are listed in Table 2. Each biological sample was run in triplicate.

**Table 2:** Sequences of the primers ^**[25, 26]**^ used for quantitative RT-PCR analysis

Strain	Forward (5′–3′)	Reverse (5′–3′)
*Enterococcus* genus	CCCTTATTGTTAGTTGCCATCATT	ACTCGTTGTACTTCCCATTGT
*Enterobacteriaceae* family	CATTGACGTTACCCGCAGAAGAAGC	CTCTACGAGACTCAAGCTTGC
*Clostridium coccoides*	AATGACGGTACCTGACTAA	CTTTGAGTTTCATTCTTGCGAA
*Lactobacillus* genus	AGCAGTAGGGAATCTTCCA	CACCGCTACACATGGAG
*Bifidobacterium* genus	TCGCGTC(C/T)GGTGTGAAAG	CCACATCCAGC(A/G)TCCAC
*E. coli*	CATGCCGCGTGTATGAAGAA	CGGGTAACGTCAATGAGCAAA
Total eubacteria (16S rRNA)	CGGTCCAGACTCCTACGGG	TTACCGCGGCTGCTGGCAC

### Statistical analysis

Data was analyzed by one-way analysis of variance, expressed as mean values ± SEM. All statistical analyses were performed by using SPSS (Version 17.0, SPSS Inc., Chicago, IL, USA). Each pig was the experimental unit. Differences among treatment means were determined by Duncan’s multiple comparison test. Additionally, differences in the means of blood biochemical and hematological parameters on day 10 of trial and the growth performance (ADG, F/G ratio and DR) was determined by the Student’s paired *t* test. Possibility values < 0.05 was considered statistically significant.

## Data Availability

The datasets used and/or analysed during the current study are available from the corresponding author on reasonable request.
